# Case of Junctional Rhythm in the Setting of Acute Adrenal Insufficiency

**DOI:** 10.7759/cureus.27605

**Published:** 2022-08-02

**Authors:** Priya Patel, Kyle Kelschenbach

**Affiliations:** 1 Internal Medicine, Florida Atlantic University, Boca Raton, USA

**Keywords:** hydrocortisone, hypotension, distributive shock, junctional rhythm, primary adrenal insufficiency

## Abstract

Primary adrenal insufficiency occurs when the production of glucocorticoid and mineralocorticoid hormones from the adrenal cortex decreases. Cardiovascular manifestations, although a rare sequela in acute adrenal insufficiency, include arrhythmias, heart failure and ischemia. Rapid identification and treatment are crucial as mortality can occur rapidly. We present a patient with no underlying adrenal dysfunction who presented with worsening renal function and subsequent development of acute adrenal insufficiency manifesting with hypothermia, hypotension, and junctional rhythm requiring vasopressor support along with hydrocortisone therapy.

## Introduction

Primary adrenal insufficiency (AI) is a condition in which there is decreased production of glucocorticoid and mineralocorticoid hormones from the adrenal cortex [1] with an incidence of 4.7 to 6.2 per million people. Classical findings of acute AI include anorexia, nausea, vomiting, and abdominal in addition to hyponatremia occurring in 90% of cases and hyperkalemia arising in 65% of cases [2-4].

Diagnosing acute AI can be challenging as the presentation of symptoms can be vague, and an acute decompensation can send individuals spiraling into circulatory shock and nonresponsive to vasopressors, leading to a rapid decline and even death [5]. Cardiac arrhythmias that present as a result of acute AI require close attention and monitoring.

This case was presented as a digital poster at the 2022 Society of Internal Medicine Southern Region Virtual Meeting.

## Case presentation

The patient is a 63-year-old male with a past medical history of depression, anxiety, multiple falls in the setting of drug abuse and overuse, delirium, chronic osteomyelitis, and hypothyroidism who presented to the emergency department for worsening renal function. During his previous admission, he was diagnosed with an acute exacerbation of his chronic osteomyelitis of the left lower extremity. Cultures were done at that time with sensitivities and the patient was discharged on piperacillin-tazobactam. His outpatient labs on follow-up showed creatinine of 7.6 mg/dL (baseline 0.7-1.1 mg/dL) and he endorsed diffuse, dull, non-radiating abdominal pain of two-day duration. His creatinine was at baseline two weeks prior to presenting to the hospital and initially, on admission, the acute kidney injury was thought to be due to piperacillin-tazobactam. 

He denied dysuria but endorsed increased volume and frequency of urination prior to arrival. He did not experience fever, chills, chest discomfort, shortness of breath, lower extremity edema, diarrhea, or constipation. He denied ingesting any alcohol, toxins, or non-prescribed supplements. No smoking or illicit drug use.

**Figure 1:**
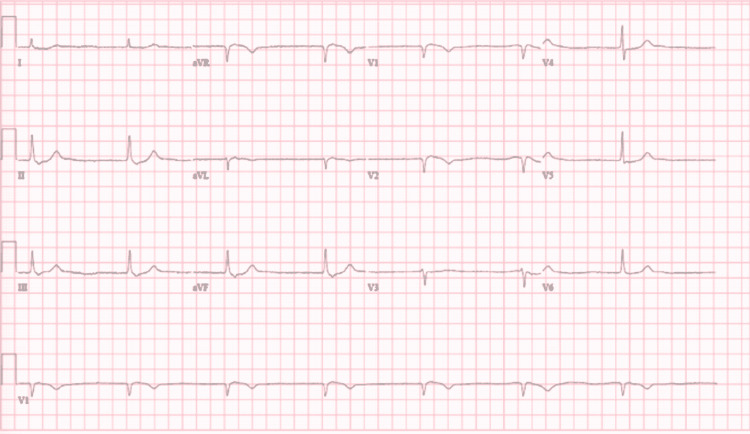
ECG tracing showing junctional bradycardia

He was one day into his hospitalization when he started becoming more confused and lethargic. The following day, he had a sudden turn of events where he became hypotensive, hypoglycemic, hypothermic, and found to be in junctional bradycardia (Figure 1).

He was transferred to the intensive care unit for further evaluation and management of shock with suspicion of possible acute AI. Norepinephrine, hydrocortisone, and levothyroxine were initiated. His lab work was significant for elevated potassium, and high thyroid-stimulating hormone (TSH) with normal thyroxine level (Table 1). His random cortisol level was low normal and the post adrenocorticotropin hormone (ACTH) stimulation test revealed a high cortisol level (Table 1) confirming the etiology of his shock as acute AI.

**Table 1:** Patient's laboratory values upon admission to the ICU

Laboratory Parameters (units)	Patient Value	Reference Range
TSH (mIU/mL)	8.1	0.4-4.6
Thyroxine (ng/dL)	1	0.61-1.12
Potassium (mEq/L)	5.9	3.5-5.2
Random Cortisol (mcg/dL)	4.2	3-10
Cortisol post ACTH (mcg/dL	23	18-20

**Figure 2:**
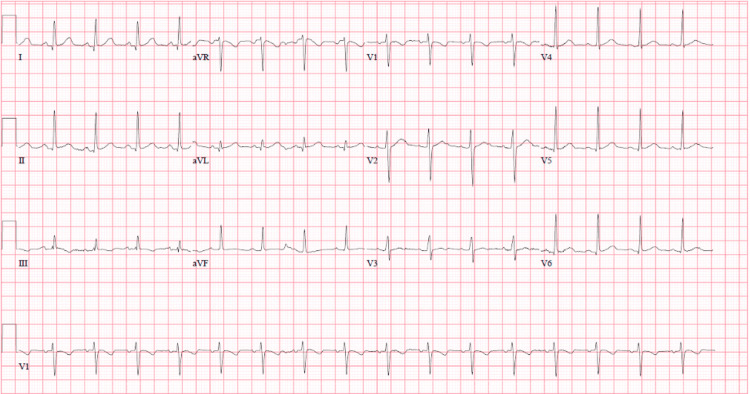
ECG tracing showing resolution of junctional bradycardia

When the patient was initially brought to the ICU his core temperature was 27 degrees Celsius and was placed on a Bair Hugger. He remained in junctional bradycardia and had a transvenous pacer placed. Over the next five days, he remained on hydrocortisone and levothyroxine therapy with daily improvement in his core temperature, hypotension, and junctional bradycardia back to a normal sinus rhythm (Figure 2). After five days of intravenous treatment, he was transitioned to oral hydrocortisone and levothyroxine. The external warming device and transvenous pacer were removed. For his acute renal failure, he was started on continuous renal replacement therapy and eventually transitioned to hemodialysis.

## Discussion

Primary AI also known as Addison’s disease, is commonly precipitated by autoimmune adrenalitis which accounts for almost 90% of cases. Other causes include infection, drug-induced, genetic factors, and acute kidney injury which is a rare cause with unknown incidence [2,3].

The unique aspect of this case is the manifestation of junctional rhythm in the setting of acute AI. The patient had no prior history of coronary artery disease, myocardial infarction, atrioventricular (AV) blocks, or bundle branch blocks nor was he prescribed any beta blocker or calcium channel blocking medications [6].

Cardiovascular manifestations of primary AI include arrhythmias, congestive heart failure, and rarely ischemic heart disease [7,8]. Cardiac arrhythmias can be related to electrolyte abnormalities associated with AI such as hyperkalemia [8]. Reports have also discussed an association of AI with dilated cardiomyopathy [9]. Our patient’s transthoracic echocardiogram showed normal left ventricular function with preserved ejection fraction. Case reports have also been published describing the development of Torsades de Pointes with hypopituitarism with complete resolution after treatment with steroids and thyroid hormone [7]. Other case reports have described severe junctional bradycardia [10] and symptomatic complete heart block [11] in the setting of acute AI, both resolving after steroid therapy.

The pathophysiology of cardiac involvement in primary AI in animal studies has shown the permissive effects of glucocorticoids on cardiac myocytes in response to epinephrine and angiotensin II that antagonize the sympathetic nervous system receptors [12,13]. Other proposed mechanisms include the direct iontropic effect of glucocorticoids, stimulation of epinephrine synthesis in the adrenal medulla and hyperkalemia leading to bradyarrhythmia [13,14].

Once out of the acute phase, individuals with primary adrenal insufficiency will require lifelong glucocorticoid therapy. These individuals have a notable increase in cardiovascular disease. The utilization of hydrocortisone at doses greater than 20mg as treatment of AI has a higher prevalence of metabolic risk factors such as obesity, diabetes mellitus and hypertension [15]. In a case control study of about 51,000 patients who received a prescription for systemic or non-systemic steroids, Soverein et al. showed there was increased cardiovascular risk when compared to matched controls versus those without steroid intake [16].

## Conclusions

AI can often present very suddenly with subtle findings requiring a high index of suspicion and a multidisciplinary approach to act promptly. Delayed identification or diagnosis is associated with higher mortality. Cardiovascular manifestations are rare and can include findings like junctional bradycardia.
